# The Impact of Fermentation Temperature and Cap Management on Selected Volatile Compounds and Temporal Sensory Characteristics of Grenache Wines from the Central Coast of California

**DOI:** 10.3390/molecules28104230

**Published:** 2023-05-22

**Authors:** Emily S. Stoffel, Taylor M. Robertson, Anibal A. Catania, L. Federico Casassa

**Affiliations:** 1 Food Science & Nutrition Department, California Polytechnic State University, San Luis Obispo, CA 93407, USA; esstoffe@calpoly.edu; 2 Wine & Viticulture Department, California Polytechnic State University, San Luis Obispo, CA 93407, USA; trober24@calpoly.edu; 3 Centro de Estudios de Enología, Estación Experimental Agropecuaria Mendoza, Instituto Nacional de Tecnología Agro-pecuaria (INTA), San Martín 3853, Mendoza 5507, Argentina; acatania@fca.uncu.edu.ar

**Keywords:** red wine, fermentation temperature, cap management, punch downs, descriptive analysis, temporal check-all-that-apply, astringency, retronasal aroma, volatile chemistry

## Abstract

Grenache wines from the Central Coast of California were subjected to different alcoholic fermentation temperature regimes (Cold, Cold/Hot, Hot) and cap management protocols, namely, punch down (PD), or no punch down (No PD), to determine the effect of these practices on the color, aroma, and the retronasal and mouthfeel sensory characteristics of the resulting wines. Descriptive analysis (*n* = 8, line scale rating 0–15) results indicated that the combination of a hot fermentation temperature and no punch downs led to a significantly higher intensity in perceived color saturation (7.89) and purple hue (8.62). A two-way analysis of variance (ANOVA) showed that cap management was significantly more impactful on the perception of orthonasal aromas than fermentation temperature. The reduction aroma was significantly higher in No PD wines (5.02) compared to PD wines (3.50), while rose and hot aromas had significantly higher intensity perception for PD wines (5.18, 6.80) than for No PD wines (6.80, 6.14). Conversely, analysis of selected volatile compounds indicated that fermentation temperature was more impactful than cap management regime. Cold/Hot wines had higher concentrations of important esters such as ethyl hexanoate (650 µg/L) and isoamyl acetate (992 µg/L). Cold wines had a higher concentration of β-damascenone (0.719 µg/L). TCATA evaluation (*n* = 8) indicated that Cold/Hot PD wines had a significantly higher citation proportion of fruit flavor (1.0) and velvet astringency perception (0.80) without significant reduction flavors. Finally, the present study represents a contribution with the main volatile compounds (e.g., β-damascenone and esters in the Cold and Cold/Hot fermented wines, respectively; hexanol in PD wines, which may be potentially responsible for a hot mouthfeel), and sensory characteristics (red fruit, tropical fruit, white pepper, and rose) of Grenache wines grown in the Mediterranean climate of the Central Coast of California.

## 1. Introduction

Grenache has proven to be extremely versatile from a winemaking viewpoint as it can be used in rosé, blended red wines (i.e., GSM blends), and monovarietal wines [1]. Grenache is considered a Mediterranean variety that produces wine of low color, tannins, and perceivable acidity [2,3]. However, its wines have a complex aromatic composition based on esters, terpenes, and even thiols [4]. Because of this, Grenache wines have been the subject of extensive research in volatile chemistry through gas chromatography-mass spectroscopy (GC-MS) and gas chromatography-olfactometry (GC-O) analysis as well as sensory assessment, typically through descriptive analysis [5,6,7]. However, there have been no studies to the authors’ knowledge that have utilized a dynamic sensory method to assess the retronasal profile of Grenache wines.

Time-based sensory methods have become popular in wine research as they are able to capture how a wine’s retronasal and mouthfeel sensations change over time. One of the newer temporal sensory methods is a rapid method known as Temporal Check-All-That-Apply (TCATA). TCATA is a time-based version of check-all-that-apply (CATA) [8]. CATA is a rapid method that requires panelists to check all the attributes they are perceiving in a sample [9]. CATA can be used to assess both orthonasal and retronasal aromas, but it is limited to assessment at a single time point. Hence, TCATA was developed utilizing the principles of CATA but allowing for a continuous and dynamic assessment of sensory properties.

TCATA shares similarities to an earlier sensory method known as Temporal Dominance of Sensations (TDS) and the two methods have been compared extensively [8,9,10,11]. Briefly, TDS relies on the notion of dominance or *“the most attention-grabbing sensation at each moment”* which limits the panelist to selecting one attribute at a time [12]. The limitation of one dominant attribute can lead to data loss for other attributes present and of interest to the researcher. Additionally, the dominant attribute decision process can increase the possibility of dithering or, inability to decide on an attribute. Dithering can lead to a lag time in the sensory profile data [9]. These limitations are made up for in TCATA, as the panelist can select multiple attributes at a time and are not restricted by the definition of dominance [8]. However, the two methods are similar in that the ideal number of attributes is between six and ten which lessens the probability of dumping [13]. TCATA is a rapid method, meaning it requires little training (less than five hours total), and has been used in consumer panels [14]. Additionally, TCATA data are converted to curves that show the citation proportion of each attribute over time which are similar to TDS curves. However, TCATA curves do not contain calculated chance and significance lines showing statistical significance in the curves. The data can be subjected to difference curves as done with TDS to determine any statistical significance between treatments [15]. While TCATA does make up for some of the main limitations of TDS, it does have its own limitations. The main limitation of TCATA is that panelists can be slower in checking and unchecking all the applicable attributes causing a lag time in the resulting TCATA curves [16].

Although TCATA is a newer method, it has been used consistently with retronasal aroma and mouthfeel studies in wine [17,18,19]. For example, Syrah wine was made with two levels of ethanol, high (15%) and low (10%), and analyzed using TCATA for in-mouth sensory properties [17]. Results indicated that the high ethanol Syrah had a significantly longer finish compared to the low ethanol Syrah from the TCATA evaluation. Additionally, a principal component analysis found that the high ethanol Syrah was associated with bitterness, astringency, and dark fruit, whereas low ethanol Syrah was strongly associated with acidity, red fruit, and green character. Another study focused only on the evaluation of mouthfeel characteristics in Pinot noir, Cabernet Franc, and Cabernet Sauvignon [19]. Four mouthfeel characteristics were evaluated by two sensory panels using TCATA. Results showed that Pinot noir had the lowest citation proportion in perceived astringency compared to the other wines. However, none of these studies focused on understanding the temporal impact of a particular winemaking technique using TCATA. Only one study, to the authors’ knowledge, has used TCATA to understand changes in a winemaking technique. This study looked at the effect of carbonation level due to the addition of different levels of dextrose in the *liqueur de tirage* in Chardonnay-based sparkling wines from the Columbia Valley, WA (USA) [18,20]. TCATA results indicated that an increase in carbonation (CO_2_ g/L) led to a longer retronasal and mouthfeel finish [18]. However, in this study, TCATA was used to evaluate a specialized form of winemaking, as such, the results are not applicable to still red winemaking.

Understanding the impact of winemaking techniques on the retronasal and mouthfeel characteristics is important especially as consumers primarily focus on this aspect of wine when making a hedonic judgment about wine [21]. From a consumer perspective, sensory characterization is the first step in understanding how a product will be perceived by the public [22]. Two of the most important technical decisions prior to the onset of winemaking for red varietals are the fermentation temperature during alcoholic fermentation and cap management regime during skin contact (i.e., maceration) time.

The choice of fermentation temperature affects the rate of alcoholic fermentation, the evolution and diversity of yeast populations, phenolic extraction, and the sensory characteristics of the resulting wine [23,24,25]. Phenolic extraction during red winemaking is particularly sensitive to fermentation temperature which in turn influences the color, taste, and mouthfeel of the resulting wine [26]. A review of winemaking techniques on phenolic extraction reported that hot fermentation temperatures (25 °C to 30 °C) favored higher phenolic extraction thus resulting in wines with higher astringency and color saturation [23]. Similarly, a study was conducted on three different clones of Pinot noir and three different fermentation temperatures, including cold (10 °C), hot (25 °C), and variable (seven days at 10 °C, and seven days at 25 °C). Results reported that a hot fermentation temperature led to wines with higher total phenolics and higher red hue according to International Commission on Illuminations (CIE) Lab results [27]. In Petit Verdot wines, fermentation at three different temperatures (17 °C, 21 °C, and 25 °C) led to significant increases in total anthocyanins and flavonols whereby color intensity increased significantly when wines were fermented at 25 °C compared to 17 °C [28].

In terms of sensory implications, a study in Syrah examined the impact of three fermentation temperature regimes (15 °C, 20 °C then 30 °C, and 30 °C then 20 °C) as well as the use of cold-soak or extended maceration [29]. Results from a pair-comparison sensory test showed that a warmer fermentation temperature (30 °C) led to a higher perceived red color and black currant flavor compared to a cold fermentation temperature (15 °C). These results suggested that a hot fermentation temperature led to a higher red hue and overripe fruit flavors. Furthermore, another study examined the impact of cold (15 °C) and control (25 °C) fermentation temperatures and cold-tolerant yeast strains in Merlot. It was reported that the cold treatments regardless of yeast strains had significantly higher concentrations of total esters, but lower total terpenes compared to the control (25 °C) [30]. The results seen in the volatile chemistry corroborated with descriptive analysis data, whereby control treatments were inversely related to the fruit aromas (red fruits and banana) according to a principal component analysis, suggesting a colder fermentation temperature led to more fresh fruit aromas.

Cap management techniques during alcoholic fermentation also have critical implications for the chemical and sensory aspects of red wines. The cap refers to the mass of skins and seeds that form at the top of the fermentation vessel due to the release of carbon dioxide. The resulting cap is typically integrated into the fermenting juice by various cap management practices. The most prevalent cap management methods applied during red winemaking are pump-overs, submerged caps, punch downs, and rack-and-return (i.e., délestage). There is extensive literature on the chemical impact of cap management techniques in red wines [31,32]. However, research on the sensory impact of these practices remains limited. One such study examined the chemical and sensory impact of punch downs, submerged caps, and an eight-week extended maceration in Merlot wines [33,34]. Descriptive analysis results showed that the punch-down wines had the lowest astringency intensity score [34]. Additionally, TDS was utilized to understand the temporal sensory impact of cap management practices [33]. Results indicated that while descriptive analysis showed differences in astringency intensity; the dominance of astringency in the TDS curves was similar among all treatments except for the submerged cap at week 0 of the treatment. However, the TDS evaluation results were limited as they did not include retronasal aromas.

When utilizing time-based sensory methods to evaluate wines, it is important to consider the confounding factor of salivary flow rate. This is because the temporal perception of astringency, retronasal aromas, and tastants (bitterness) can be affected by the salivary flow rate. Previous research has indicated that individuals with a low salivary flow rate perceive astringency later and more intensely than high salivary flow rate individuals [35,36,37]. The same pattern has been seen with bitterness perception [36].

The relationship between retronasal aromas and salivary flow rate appears to be based on the aroma type and its corresponding volatile compounds. A study was conducted on Syrah with spiked volatile compounds (isobutyl methoxypyrazine, phenylethyl alcohol, and lactone) corresponding to vegetal, floral, and coconut aromas, respectively. It was determined that individuals with high salivary flow rates experienced the intensity of these compounds significantly later and more intensely compared to low salivary flow rate individuals [38]. As for fruit aromas, which are associated with esters, a study that examined commercial Tempranillo and Verdejo rosé wines that were spiked with four esters (isoamyl acetate, ethyl butanoate, ethyl hexanoate, and ethyl decanoate) determined that the short chain esters were perceived significantly more intensely by high salivary flow rate panelists than low salivary flow rate panelists [39].

Common to all these studies was the use of the temporal sensory method known as Time-Intensity (TI). This method is like descriptive analysis (DA) in that it uses a 10 or 15 cm scale to assess the intensity of an attribute. However, TI assesses the intensity of one or two attributes over time typically after the wine has been expectorated [40]. A recent study examined the effect of salivary flow rate and the perception of astringency, bitterness, and mineral retronasal aromas utilizing TDS in red wines that had been co-fermented or blended with a combination of two or three varietals (Merlot, Malbec, and Petite Sirah) [41]. The results of the study found the same trends between salivary flow rate and the time to onset of dominance for astringency and bitterness as seen in past studies with TI and intensity assessment. Individuals that were considered to have low salivary flow rates experienced the time to onset of dominance for astringency and bitterness later than the high salivary flow rate panelists. However, no studies to the authors’ knowledge have utilized TCATA to further understand salivary flow rate and time of perception.

Overall, there is a scarcity of research examining the sensory and volatile composition of Grenache wines that have undergone different alcoholic fermentation temperature and cap management regimes. Therefore, in the present study, Grenache wines from the Central Coast of California (USA), were produced in triplicate fermentations by a combination of three contrasting fermenting temperature regimes (Cold: 12 °C; Cold/Hot: seven days 12 °C, and seven days at 28 °C; Hot: 28 °C) and two contrasting cap management regimes, punch down (PD) and no punch down (No PD). The objective of this study was to understand the impact of fermentation temperature and cap management on the detailed sensory and volatile composition of Grenache wines. A combination of descriptive analysis and TCATA was used to assess the temporal retronasal impact. Selected volatile compounds were analyzed in the wines to assess the relationship between volatile chemistry and sensory perception. An additional purpose of the study was to expand the understanding of the relationship between salivary flow rate and time of perception for selected retronasal and mouthfeel sensations. 

## 2. Results and Discussion

### 2.1. Perceived Color

Two color properties (saturation and purple hue) were assessed to understand the effects of fermentation temperature (Cold, Cold/Hot, and Hot) and of two cap management styles (PD and No PD) in Grenache wines. Results for color and aroma attributes are shown in Table 1. Both color attributes, saturation (*p* = <0.0001) and purple hue (*p* = <0.0001), were statistically significant. Hot No PD wines had significantly higher color intensity compared to all other wines, including the Hot PD wines. Cold No PD wines were significantly lower in saturation compared to all other wines. According to a two-way ANOVA, fermentation temperature was statistically significant for saturation (*p* = <0.0001) with Hot wines significantly higher than all other fermentation temperatures (Supplemental Table S1). The factor of cap management on its own was not significant but there was a significant interaction between the two factors for saturation (*p* = <0.0001).

To date, there have not been many studies that have examined the sensory impact of fermentation temperature on perceived color saturation. Results of past studies have shown that an increase in fermentation temperature led to increased color properties from a chemical perspective [24]. For example, a study conducted on Pinot noir which was fermented at 20 °C, 30 °C, and using a high intensity/short time heat treatment (90 °C to 95 °C) found that concentration of polymeric pigments had a strong, inverse correlation to the lightness (L*) parameter in CIE Lab measurements, meaning the wines were darker in saturation [42]. Another study examined the impact of flash release (heating fermenting musts 95 °C or more) on the resulting phenolic composition of Grenache, Mourvèdre, and Carignan wines over two vintages [43]. The application of flash release led to higher levels of anthocyanins during fermentation. However, after alcoholic fermentation, flash-release wines saw a decrease in anthocyanins and an increase in tannin to anthocyanin ratio. It is important to note that this study was limited in that it examined anthocyanin, tannins, and total polyphenol index, but the relationship between heat during fermentation and polymeric pigment formation was not examined. Findings from the previous literature align with the chemical results of the present study. Herein, Hot wines regardless of cap management procedure had significantly higher total polymeric pigments (*p* = <0.0001) compared to the other fermentation temperatures (Table 2). For anthocyanins (*p* = 0.001), both Cold wines and the Hot PD wines were significantly lower compared to Hot No PD wines and both Cold/Hot wines which suggested a potential influence of cap management. Both total anthocyanins (*p* = 0.001) and total polymeric pigments (*p* = <0.0001), were statistically significant for the factor fermentation temperature, but not for the factor cap management (Supplemental Table S2). Additionally, total polymeric pigment concentration showed a positive correlation to perceived color saturation whereby Hot wines had significantly higher saturation. However, there was a significant interaction between cap management and fermentation temperature for both parameters, therefore it was likely cap management had some influence on color extraction. A potential explanation for the interaction between fermentation temperature and cap management could be a mixing effect proper of alcoholic fermentation, whereby carbon dioxide naturally mixes the bottom of the fermenting cap and promotes phenolics extraction [31]. This explanation was suggested by a previous experiment that examined the impact of pump-over volume and frequency in Cabernet Sauvignon [31].

Purple hue intensity also increased with fermentation temperature. Significant differences were apparent for purple hue (*p* = <0.0001) between fermentation temperature regimes but not cap management (Table 1). Hot wines had a significantly higher purple hue compared to both Cold/Hot and Hot wines. Cold wines had a significantly lower purple hue compared to the other fermentation temperatures. The same trend was seen in the two-way ANOVA but the interaction between fermentation temperature and cap management was statistically significant (*p* = <0.0001) (Supplemental Table S2). Previous research on color as affected by alcoholic fermentation temperature primarily focused on the red hue, likely because the red hue can be directly assessed through the red/green chroma (a*) parameter in CIE Lab analysis. For example, Pinot noir wines were fermented at Cold (10 °C), Hot (25 °C), and Variable (10 °C then 25 °C) temperature regimes; resulting CIE Lab analysis showed that the Hot wines led to significantly higher a* levels or red hue [27]. The use of red hue has also been seen in sensory tests in the literature. A pair-comparison test on Syrah wines that were fermented at 15 °C and 30 °C and received cold soak, extended maceration, or neither, determined that wines with increased fermentation temperature led to more red color [29]. However, it appears that in terms of fermentation temperature purple hue in the present study showed similar behavior to that seen in red hue from past research. Overall, in the present study, a hot fermentation temperature and No PD led to wines with higher color saturation and purple hue.

### 2.2. Orthonasal Aroma Attributes

**Figure 1:**
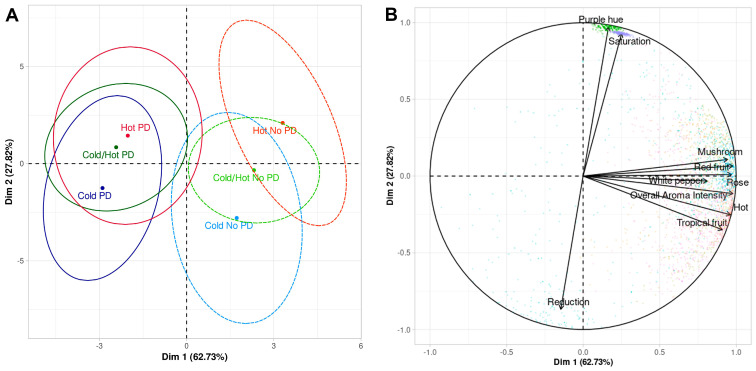
Principal component analysis of descriptive analysis data Grenache wines evaluated by a trained sensory panel (*n* = 8). (**A**) Treatment confidence ellipses constructed using Hotelling’s T2 test (*p* < 0.05) for each pair of products indicate 95% confidence intervals. (**B**) Sensory attribute loadings.

A principal component analysis (PCA) was conducted on the descriptive analysis data and calculated using Dimension 1 (eigenvalue: 6.27; variability: 0.627) and Dimension 2 (eigenvalue: 2.78; variability: 0.278) (Figure 1A,B). The resulting PCA explained 90.6% of the variation within the dataset. Dimension 1 separated wines by cap management regime while Dimension 2 separated the wines by fermentation temperature whereby Cold/Hot and Hot wines were distinguished from Cold wines. Additionally, the No PD wines appeared to be more correlated with most of the sensory loadings compared to the PD wines (Figure 1A,B). The stronger correlation between most of the sensory characteristics and No PD wines may be in part due to the sensitivity of Grenache wines to oxidation [7]. The latter has been attributed to the high concentration of hydroxycinnamic acids (270–460 mg/L caftaric acid) in Grenache wines compared to other varieties (50–60 mg/L caftaric acid) as these compounds assist in the oxidation process of wine [1,43]. Therefore, it seems plausible that excessive cap management may lead to lower aroma retention in Grenache wines due to oxidation. Indeed, a previous study examined the effect of oxygen exposure on the sensory profile of bottled Sangiovese, which is another red variety that is highly susceptible to oxidation [44]. Results indicated that after 10 months of oxygen exposure, wines had significantly higher balsamic aromas (indicative of oxidation) over other aromatic attributes.

Interestingly, according to the one-way ANOVA, the reduction aroma (*p* = <0.0001) was the only statistically significant attribute (Table 1). For reduction (*p* = <0.0001), both the Cold and Hot wines showed statistically significant variation between cap management regimes (Table 1). Cold No PD wines were significantly higher in reduction aroma than Cold PD wines; the same trend was observed between the Hot No PD wines and Hot PD wines. In the two-way ANOVA, the No PD wines regardless of fermentation temperature were significantly higher in reduction aromas (Supplemental Table S1). The No PD wines also had a significantly greater mushroom aroma (*p* = 0.0019). The aromas of reduction and mushroom are associated with hydrogen sulfide, methanethiol, and other volatile sulfur compounds [45,46]. The formation of reduction-associated aromas, particularly during alcoholic fermentation, can be attributed to a lack of, or suboptimal oxygen exposure to the fermenting must, among other factors. For example, a study conducted on Shiraz to understand the effect of oxygen exposure on volatile sulfur compounds production showed that wines that were sparged with air (20% O_2_) or a 40% oxygen-nitrogen mixture had significantly less hydrogen sulfide compared to a control (no gas sparging) or nitrogen sparging post-inoculation [47]. Sensory results corroborated with the volatile chemistry, whereby the control and nitrogen sparging treatments were strongly associated with the aromas attributes rotten egg, sewage, rubber, and non-fruit persistence which were inversely related to the air (20% O_2_) and 40% oxygen-nitrogen mixture wines.

During alcoholic fermentation, oxygen exposure to the wine can occur during cap management procedures such as punch downs or pump-overs. Therefore, it stands to reason that the No PD wines of the present study had significantly higher reduction aromas compared to the PD wines as no cap management occurred in the No PD wines. It is important to note that a lack of cap management is not the only factor that can contribute to the reduction of related aromas in red wines. Other factors such as yeast strain, and improper yeast nutrition can exacerbate the reduction of aromas as previously shown [48]. Another important factor related to reduction aromas is fermentation temperature, whereby a warmer fermentation temperature can lead to increased reduction aromas [49]. In the present study, however, fermentation temperature did not have a significant impact on the reduction of aromas seen in the wines (Supplemental Table S1). Nonetheless, there was a significant interaction between cap management and fermentation temperature (Supplemental Table S1). This was also seen in the one-way ANOVA of reduction aromas whereby Cold No PD, Cold/Hot No PD, and Hot No PD wines had the highest reduction aroma intensity and were not significantly different from each other (Table 1). Therefore, fermentation temperature likely had some influence on the resulting sensory profile of these wines. It is also important to note that sulfur-based compounds were not measured during volatile compound analysis of the present study (Table 3), as sulfur compounds require very specific methods of analysis for detection [50,51].

**Figure 2:**
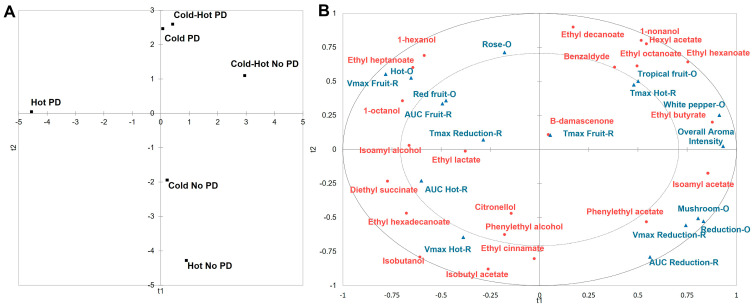
Partial least square regression analysis of volatile chemistry and sensory attributes. (**A**) Wine treatments (■). (**B**) Correlation loading of the relationship between the volatile chemistry (●) and sensory attributes (▲). O: indicates orthonasal aroma; R: indicates retronasal aroma; V_max_: maximum citation proportion; T_max_: time of maximum citation proportion; AUC: area under the curve, total intensity response.

Overall aroma intensity and three aroma attributes, namely rose, tropical fruit, and white pepper did not have statistically significant *p*-values, but these attributes showed variation in the *post-hoc* test in the one-way ANOVA (Table 1). In the two-way ANOVA, the PD wines were significantly higher in rose (*p* = 0.031) and hot (*p* = 0.018) aromas (Supplemental Table S1). Previous reports suggest that the rose aroma in red wines and particularly Grenache wines can be circumscribed to three different classes of volatile compounds including esters (phenylethyl acetate), terpenes (citronellol), and alcohols (phenylethyl alcohol) or a combination of them [61,62]. Indeed, a previous study, which examined the sensory and volatile chemistry of Cannonau (Grenache) from different regions in Sardinia (Italy), found that the floral aromas were strongly associated with citronellol and isoamyl acetate while floral flavors were associated with phenylethyl alcohol and limonene [5]. This was not the case in the present study. Both phenylethyl acetate and citronellol had odor activity values (OAVs) lower than one and, therefore, were unlikely to be of sensory relevance (Table 3). Phenylethyl alcohol did have OAVs higher than one for all wines and had its concentration significantly effected by fermentation temperature with a significant interaction with cap management (*p* = <0.0001) (Table 3, Supplemental Table S3). A Partial Least Square Regression (PLSR) analysis was run to relate volatile chemistry with sensory descriptors. The PLSR model, which was calculated using Factor 1 (R^2^ X: 0.33; R^2^Y: 0.44; Q^2^: −0.07) and Factor 2 (R^2^ X: 0.65; R^2^Y:0.71; Q^2^: 0.13), explained 71.2% of the variation in the dataset, showing that the rose aroma was not strongly associated with phenylethyl alcohol (Figure 2A,B). It is then possible that the rose aroma could have been attributed to a volatile compound that was not detected in the GC-MS methods used. Alternatively, this aroma could be due to the wine aroma buffer which is a combination of several volatile compounds in wine that contribute to its “vinous” aroma character [61].

As for the hot aroma, in the present study, it was defined as *“the aromatic burn of ethanol”.* Hot aromas in wine are typically associated with higher levels of ethanol. Empirically, monovarietal Premium or Ultra-premium Grenache wines produced commercially tend to be wines with 14.5 to 15.5% ethanol (*v*/*v*). However, this was not the case for the wines of the present experiment, whereby ethanol in the finished wines ranged from 12.8% to 13.3% ethanol (*v*/*v*) (Table 4). While the one-way ANOVA showed variation in the *post-hoc* test, the two-way ANOVA indicated that ethanol was not significantly impacted by fermentation temperature, cap management, or their interaction (Supplemental Table S4). Three factors might explain this discrepancy. First, even though panelists did detect the burn of ethanol in all the wines, they did so at a moderate level (based upon an average rating of 6.5 sensory units over 15). Second, it is possible that through cross-modal association [63] panelists might have ascribed red fruit and tropical fruit aromas to heat which is otherwise a character sometimes associated with Grenache wines from Mediterranean climates. Third, and most likely, the perception of the hot aroma could have been caused by the volatile compound 1-hexanol. 1-hexanol is a C6 alcohol attributed with herbaceous and resinous aroma characteristics [64]. 1-hexanol showed extremely high OAV values, of three orders of magnitude, in all wines indicating the compound had high sensory relevance (Table 3). In the two-way ANOVA, 1-hexanol (*p* = <0.0001) was significantly higher in PD wines compared to No PD wines (Supplemental Table S3). As well, 1-hexanol and the hot aroma were closely related in the PLSR (Figure 2). This aligns with the results of the previous literature, whereby the volatile and phenolic chemistry impact of a floating cap treatment compared to submerged cap treatment in Barbera wines were examined [65]. Results indicated the floating cap treatments combined with pump overs had a significantly higher concentration of hexanol than the submerged cap wines, which was thought to be due to enzymatic oxidation.

The only aroma attribute influenced by fermentation temperature was white pepper which was higher in the Cold and Cold/Hot wines. However, intensity ratings of white pepper aroma were low, ranging from 2.80 to 3.51 in scores on a 15 cm line scale (Table 1), so this specific attribute was unlikely to have a strong sensory impact.

All the wines were similar in overall aroma intensity as well as red fruit, and tropical fruit aromas regardless of treatment. An explanation for the overall lack of differences seen with aroma intensities between the wines may have been due to a masking effect caused by reduction aromas and their associated chemical compounds. Two compounds responsible for reduction aromas in wines are hydrogen sulfide and methanethiol [46]. A previous study examined the aromatic impact of these two compounds as well as their interaction in model white, red, and red oak wines based on typical Spanish wines [66]. Wines were evaluated by a sensory method known as Rate-All-That-Apply (RATA). The results indicated that the presence of hydrogen sulfide and methanethiol significantly repressed fruit and floral aromas in the white and red wines. Based on these findings, it is likely that a similar masking effect occurred with the Grenache wines of the present study particularly as the aroma descriptors included attributes like rose, tropical fruit, and red fruit. However, the volatile chemistry results showed differences among the wines even if they may have not been perceived from a sensory perspective. Indeed, Cold wines had high concentrations of β-damascenone and Cold/Hot wines tended to have higher concentrations of the esters ethyl butyrate, isoamyl acetate, ethyl octanoate, and ethyl hexanoate (Table 3). Hot wines also had high concentrations of isoamyl acetate (Table 3). All of these compounds with high OAVs are associated with fruit aromas and therefore they were likely masked by reduction aromas.

### 2.3. Taste, Mouthfeel, and Retronasal Aroma

Overall, the most cited attributes during TCATA evaluations were bitterness, the astringency subquality suede, and acidity as indicated by the highest maximum citation proportion (V_max_) values compared to all other attributes (Table 5). Additionally, bitterness, suede, and acidity had the highest AUC (total intensity response) values. These three attributes also had the longest length (T_first_–T_last_) with the addition of the astringency subquality velvet. Interestingly, the retronasal aromas of fruit, hot, and reduction consistently had the lowest time parameter values most notably, AUC, V_max_, and length. An exception to this trend was that the astringency subquality velvet had the lowest V_max_ value (maximum citation proportion) (Table 5). The most cited attributes of the present study reflected results seen in a past experiment that utilized TDS to examine the retronasal and mouthfeel sensations in blended and cofermented Malbec, Merlot, and Petite Sirah, whereby astringency, bitterness, acidity, and the retronasal aroma mineral were the most dominant attributes [41]. Similarly, another study examined the retronasal and mouthfeel sensations in six different red varietal wines using TDS and TCATA [67]. The TDS results indicated that acidity and astringency were the most dominant attributes, while TCATA results showed a similar pattern but with the addition of bitterness as the most cited attribute. Both past studies included retronasal aromas, but, for the most part, these retronasal aromas were not as cited or dominant depending on their respective sensory methods [41,67]. The same trend has been observed in the present study. The similarities between the results of TDS and TCATA may be an indication that panelists interpreted dominance and the presence of an attribute in the same way. Additionally, astringency, bitterness, and acidity are typically constant attributes in red wines, while retronasal aromas can change depending on the varietal and winemaking style. This means that panelists may be more confident in their perception of acidity, astringency, and bitterness. Hence, these attributes had higher citation proportions in the temporal retronasal sensory studies.

**Figure 3:**
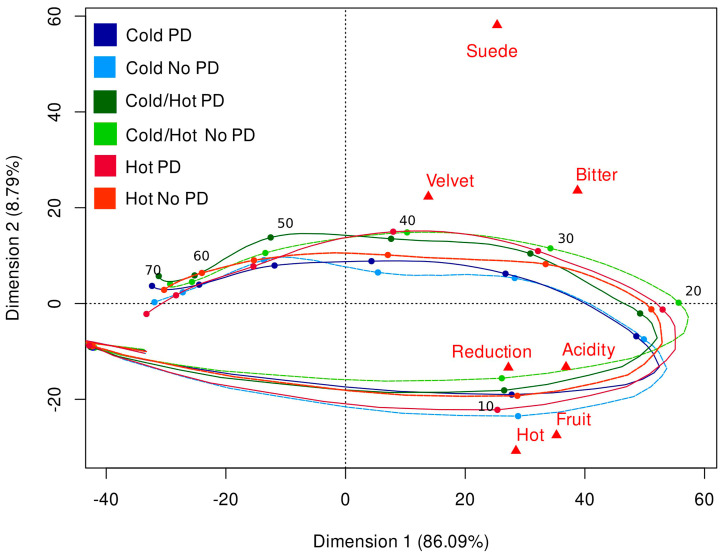
Principal component analysis for the trajectory timeline (seconds) of TCATA attributes in Grenache wines evaluated by a trained sensory panel (*n* = 8).

A PCA was conducted to show the product trajectory of each treatment and the order of attribute perception during evaluation (Figure 3). Dimension 1 (eigenvalue: 5.79; variability: 0.827) and Dimension 2 (eigenvalue: 0.76; variability: 0.108) were used to calculate the PCA and explained 95% of the variation within the dataset. The lower right quadrant was dominated by the attributes reduction, acidity, hot, and fruit, which appeared early in the evaluation at around 10 s. The upper right quadrant was dominated by the suede, velvet, and bitter attributes indicating that these attributes were perceived in the middle of the evaluation between 30- and 40-s. This order of perception has been seen previously in wine research with time-based sensory analysis [33,41,67]. However, the attribute lines are close to one another, suggesting that the retronasal and mouthfeel profiles were not largely different from each other. The exception to this trend was the Cold fermentation temperature wines which separated and were lower than the other wines from 20 to 40 s. While Figure 3 can show broad differences among wines and the order of attribute perception, it is not able to show significant differences among treatments.

**Figure 4:**
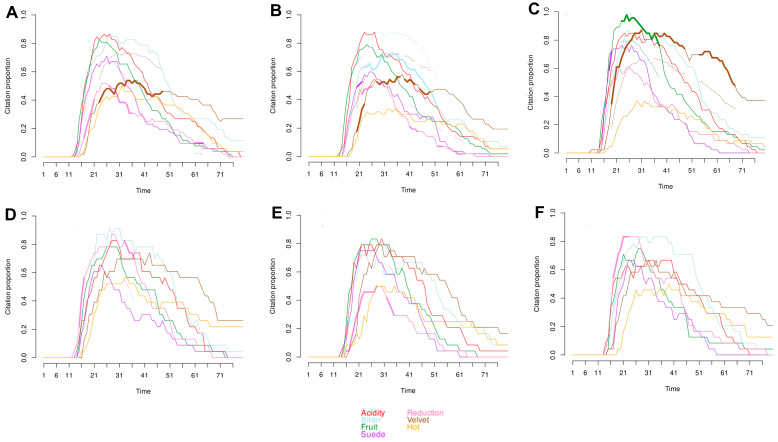
Temporal check-all-that-apply (TCATA) curves. (**A**) Cold PD; (**B**) Cold No PD; (**C**) Cold/Hot PD; (**D**) Cold/Hot No PD; (**E**) Hot PD; (**F**) Hot No PD. When attributes within each treatment are significantly different from all other wines the line is highlighted, and a dotted reference line of the same attribute indicates where the average citation proportion of the other wines is for that attribute.

The TCATA curves for each wine are shown in Figure 4A–F with highlighted lines indicating significant differences of an attribute compared to all treatments. Interestingly, while acidity and bitterness were the most cited attributes, most of the wines did not have statistically significant differences comparatively. The exception was the Cold No PD wines which had a significantly lower citation proportion of bitterness from 11 to 46 s (Figure 4B). This was expected as acidity perception is linked to titratable acidity (TA), and although there were significant differences in TA between wines, these are unlikely to be of sensory relevance (Table 4).

Similarly, suede, which also had a high V_max_ as shown in Table 5, was only significantly higher in Cold PD and Cold/Hot PD wines for a brief period (five seconds) (Figure 4A,C). Otherwise, the wines were either not statistically significant for suede or in the case of Cold No PD had a significantly lower citation proportion of suede for 5 s. As for the velvet sub-quality of astringency, both Cold wines had significantly lower citation proportions during the middle of the evaluation while Cold/Hot PD wines had significantly higher citation proportions during the middle of the evaluation. Differences in TCATA curves were reflected in phenolic composition among winemaking treatments. The Cold/Hot and Hot wines regardless of cap management had higher phenolic composition compared to the Cold wines (Table 2). The two-way ANOVA of phenolic composition indicated that Cold/Hot and Hot wines were not significantly different in terms of anthocyanins, total tannins, and phenolics (Supplemental Table S2). These results were expected as the Cold/Hot wines of the present study underwent a treatment similar to a cold soak in which the must is held at a cold temperature prior to fermentation, followed by a warm fermentation temperature [23]. However, the Cold/Hot wines were fermenting during the temperature changes. The application of cold-soak during winemaking has been shown to have no effect on the phenolic composition of wines specifically tannins, total phenolics, and anthocyanins [68]. Interestingly, the highest citation proportion and longest persistence of velvet were seen in the Cold/Hot PD wines (Figure 4C). According to a previous study that examined the chemical and sensory effect of cold soaking compared to a control in six different varietals, the use of cold soak did not significantly affect the perception of astringency [68]. However, the differences seen in the present study may be due to the definitions of astringency used which were the sub-qualities suede and velvet. These two subqualities were defined and understood by the panel as medium and low levels of astringency for suede and velvet, respectively.

The attribute that showed the most statistical variation in all the wines was reduction. Indeed, all wines except for Cold/Hot PD, had significantly higher citation proportions of reduction at some point during evaluation (Figure 4). For Cold/Hot No PD and both Hot wines, the only significantly different attribute was the reduction retronasal aroma (Figure 4D–F). Like the results seen in the orthonasal assessment of the wines, reduction was influenced by the cap management regime. This was probably due to the lack of oxygen exposure in the No PD wines during winemaking as well as the potential factors of fermentation temperature, and yeast nutrition as explained by previous research [49,69]. Although there has been research conducted on the reduction aroma in wines using gas chromatography [50,51,70], and static sensory methods [66,69,71], to the authors’ knowledge, the temporal sensory pattern of perceived retronasal reduction has not been reported before. Based on the winemaking treatments and varietal choice of the present study, the retronasal perception of reduction appears to be first perceived at a similar timepoint as bitterness which was later compared to the other retronasal aromas (Table 5; Figure 3). However, on average, the retronasal perception of reduction did not have as long of a length as the other attributes assessed; in fact, it had the second shortest length, with the shortest length belonging to hot retronasal aroma (Table 5).

Cold/Hot PD wines were the only wines that did not have a significant occurrence of reduction flavor. They also showed a significantly higher citation proportion of fruit retronasal aromas from 11 to 31 s post-expectoration which was not seen in any of the other wines. The Cold/Hot wines were similar to that of a cold-soak treatment [68]. Previous research on the application of cold-soak to Syrah wines reported an increase in black currant flavor compared to the control according to a pair-comparison test [29]. Therefore, the Cold/Hot PD treatment may have enhanced fruit flavors due to its similarity to a prefermentative cold-soak treatment. However, it must be noted that unlike traditional cold-soak regimes whereby usually no yeast is inoculated, in the present study the wines were inoculated with a selected yeast strain at the onset of skin contact time immediately post-crushing. Overall, the TCATA evaluation method provided extensive information about the wines and selected evaluation attributes.

### 2.4. Salivary Flow Rate and Temporal Sensory Perception

A one-way ANOVA conducted on the salivary flow rate for each panelist is shown in Supplemental Table S5. The mean salivary flow rate was 2.50 mL/min and there was a two-fold difference between the maximum (3.18 mL/min) and minimum (1.39 mL/min) salivary flow rate within panelists. Categorical salivary flow rates were designated based on the value’s position above or below the mean. Values above the mean were designated as high salivary flow rate (HF); while values below the mean were designated as low salivary flow rate (LF). Within the sensory panel (*n* = 8), there were six individuals with high salivary flow rate (2.53 mL/min to 3.18 mL/min; two panelists were considered low salivary flow rate (1.39 mL/min to 1.58 mL/min). Supplemental Table S6 shows a one-way ANOVA for the first time of perception by the two astringency subqualities and fruit retronasal aroma for salivary flow rate. The astringency subquality suede (*p* = 0.001) was the only statistically significant attribute, whereby LF panelists perceived it significantly later post-expectoration compared to HF panelists. Based on the present results, it appears the suede subquality may reflect the established pattern where LF individuals perceived astringency later than HF individuals which aligns with the results of previous research [35,37]. However, this panel was small in size (*n* = 8) and had many HF panelists (*n* = 6) compared to LF panelists (*n* = 2). Therefore, more assertive conclusions are not possible in light of these results. Further research with a more balanced panel in terms of salivary flow rate would be required for unequivocal conclusions.

## 3. Materials and Methods

### 3.1. Winemaking

Grenache grapes were sourced from the Rancho Real vineyard in the Santa Maria Valley AVA (Santa Barbara County, CA, USA) during the 2021 vintage. The winemaking protocol followed that of a previously published study [27], with the following modifications. 1260 kg of fruit were manually harvested and transported to Cal Poly Pilot Winery for processing the same day. Clusters were crushed/destemmed using a crusher–destemmer (Bucher Vaslin, Niederweningen, Switzerland), and resulting musts went immediately into 60 L food-grade fermenters (Speidel, Swabia, Germany), at a rate of 50 kg per vessel, measured on an analytical scale (Cardinal Detecto, Model 204, Webb City, MO, USA). Fifty mg/L of sulfur dioxide (SO_2_) was added to each fermenter immediately post-crushing. Three different fermentation temperature regimes, combined with two contrasting cap management regimes, were established: Cold (targeting an average fermentation temperature of 12 °C), Cold/Hot (fermentation temperatures of 12 °C from day 0 to 7, and 28 °C from day 8 to 14), and Hot (targeting an average fermentation temperature of 28 °C throughout). In addition, two contrasting cap management regimes per fermentation temperature regime were established, namely no punch down (No PD), whereby the fermenters received no cap management during the fermentation/maceration period; and punch down (PD), whereby fermenters received two daily manual punch downs (9 a.m. and 5 p.m.), each lasting one-minute, performed with a must plunger. All individual treatments were established in triplicate (*n* = 3), affording a total of 18 individual fermentations, of the following treatments: Cold PD; Cold No PD; Cold/Hot PD; Cold/Hot No PD; Hot PD; Hot No PD. Musts were inoculated with dry yeast (Enartis D20, 153 Windsor, CA, USA), at a rate of 40 g/hL, 6 h after crush, and fermenters were taken to their respective fermentation conditions. The selected yeast was chosen for its ability to ferment at a wide temperature range, as previously shown [27]. Total maceration time was set to 14 days.

Cold fermentation temperature treatments were established by placing the fermenters in a temperature-controlled cold room set at 10 °C for the duration of the 14-day maceration period. The Cold/Hot fermentation regime treatments were established by placing the fermenters for 7 days in a temperature-controlled cold room set at 12 °C and were then subsequently transferred to a warmer environment in a temperature-controlled room set at 26 °C, for the last 7 days of the maceration period. The Hot fermentation regime treatments were established by placing the fermenters in a temperature-controlled room set at 26 °C, for the duration of the 14-day maceration period. Temperature probes with integrated data logging software (Elitech RC-5, 8 Elitech, Milpitas, CA, USA), were placed inside each fermenter approximately 8 cm from the bottom and the probes were set at one-minute collection intervals. Plastic tubing with holes for free liquid flow was inserted vertically into the fermenters to allow for optimal probe placement in the must, below the cap, and above the bottom of the tanks, as previously described [27]. Temperature probe data was extracted using the ElitechLog software (Elitech, Milpitas, CA, USA). Diammonium phosphate (DAP) at a rate of 200 mg/L (Fermaid K, Scott Laboratories, Petaluma, CA, USA) was added on day two of alcoholic fermentation to all fermenters. Total soluble solids (°Brix), and daily temperature were monitored via a densitometer (Anton Paar DMA 35 Basic, Graz, Austria) during the duration of the 14-day maceration period, after which wines were pressed off the fermentation solids. Press fractions were discarded, with only free-run wines transferred to 18 L glass carboys. Wines were subsequently inoculated with *Oenococcus oenii* (VP41, Scott Laboratories, Paso Robles, CA, USA), at a rate of 20 g/hL to undergo malolactic fermentation. Malolactic fermentation was monitored via enzymatic analysis of L-malic acid and L-lactic acid with an enzymatic analyzer (Admeo Y15, Angwin, CA, USA), using commercial enzymatic analysis kits (Biosystems, Barcelona, Spain). Malolactic fermentation was completed in all the wines (malic acid < 0.1 g/L) (Supplemental Table S4). Cold stabilization occurred at 10 °C for 30 days, after which all wines were racked and adjusted to 30 mg/L SO_2_. The wines were filtered through 8 μm cellulose filter pads (Vintner’s Vault, Paso Robles, CA, USA), and readjusted to 30 mg/L SO_2_. The wines were bottled in early March 2022, using DIAM 5 micro-agglomerated cork closure (G3 Enterprises, Modesto, CA, USA; oxygen transmission rate; 0.4 ng/bottle/year; oxygen initial release: 1.3 mg), stored in a vertical position and kept in cellar-like conditions (approximately 12 to 14 °C) until analyses. Wines received a six-month bottle aging period prior to sensory analysis.

### 3.2. Basic Chemistry and Phenolic Measurements

pH (Thermo Scientific Orion Star A211) was measured manually. Titratable acidity (TA) was measured by titrating a known quantity of wine (5 mL) in a deionized water solution against 0.067 N NaOH (Fisher Scientific, Waltham, MA, USA) to a pH endpoint of 8.2. Ethanol (% *v*/*v*), was measured by near-infrared spectroscopy using an Alcolyzer Wine M/ME analysis system (Anton Paar, Graz, Austria). Acetic acid, glucose, fructose, malic acid, and lactic acid were determined enzymatically using commercial enzymatic analysis kits (Admeo, Biosystems Group, Hollister, CA, USA).

Phenolic compounds were analyzed using spectrophotometric methods. Wine samples were centrifuged in a 15,000× *g* microfuge (model 5415D; Eppendorf, Hamburg, Germany), and the supernatant was transferred into clean 1 mL Eppendorf tubes prior to analysis. Anthocyanins and total polymeric pigments (TPP) [the sum of small polymeric pigments (SPP) and large polymeric pigments (LPP)], were determined as previously reported [72]. Tannins were analyzed by protein precipitation [73]. Total phenolics (expressed as mg/L (+)-catechin equivalents, CE), and anthocyanins (expressed as mg/L malvidin-3-glucoside), were measured following a previously published method [72]. Non-tannin phenolics were calculated as total phenolics minus protein precipitable tannins. All spectrophotometric measurements were made with a Cary 60 UV-Vis spectrophotometer equipped with an 18-sample cell auto-sampler (Agilent Technologies, Santa Clara, CA, USA).

### 3.3. Volatile Compound Analysis

Volatile compounds for analysis were selected based on previous reports on Grenache [1,4,5]. Standards of the volatile compounds to be analyzed were purchased from Acro’s Organics (Thermo Fisher Scientific, Fair Lawn, NJ, USA), Alfa Aesar (Thermo Fisher Scientific, Fair Lawn, NJ, USA), Fischer Chemical (Thermo Fisher Scientific, Fair Lawn, NJ, USA), Sigma Aldrich (Sigma Aldrich, Sigma Aldrich Inc., St. Louis, MO, USA), Spectrum (Spectrum Chemical MFG. Corp., Gardena, CA, USA), and TCI (Tokyo Chemical Industry Co., Ltd., Portland, OR, USA). Reference compound information including CAS number, manufacturer, and purity are shown in Supplemental Table S7.

Stock solutions of volatile standards to optimize compound separation and calculate the response factor for quantification were prepared in 95% ethanol. Reference compounds were grouped according to typical concentration in wines to create thirteen stock solutions with no more than five reference compounds per stock solution and 50 µg/L of 2-undecanone (Sigma Aldrich, Sigma Aldrich Inc., St. Louis, MO, USA) which was used as the internal standard for all compounds (Supplemental Table S8) [74]. The creation of stock solutions and internal standard (ISTD) solutions were based on a previously described procedure [75]. However, stocks 11–13 required higher concentrations therefore the stock solution process was modified. For stock solution 11, ethyl hexadecanoate was added at 50 µL in 250 mL and then 115 µL was pipetted into the model wine for analysis (Supplemental Table S8). A model wine solution was utilized to optimize the separation of the volatile standards and calculate the response factor for each compound which was based on a previously described procedure [74]. The volume of each stock solution pipetted into the sample was in the middle range of the typical concentration of each compound found in wine, which varied depending on the compound, and compounds were verified using their respective qualifying ions (Supplemental Table S8). Model wine solutions were analyzed in triplicate following the analysis parameters described below for solid-phase microxextraction and stir-bar sorptive extraction procedures. 

#### 3.3.1. Solid-Phase Microextraction

Solid-phase microextraction (SPME) was used to analyze and quantify the compounds isoamyl alcohol, isobutanol, ethyl lactate, and ethyl hexadecanoate. Sample preparation, sampling, and instrument analysis followed a previously outlined procedure with modifications [74]. For each sample, 10 mL aliquot (model wine solutions or wine samples) was pipetted into 20 mL round-bottomed glass vials (Gerstel, Linthicum, MD, USA) containing 3 g of NaCl (ACS reagent, Sigma Aldrich, Sigma Aldrich Inc., St. Louis, MO, USA) and 6.18 µL of 2-undecanone (Sigma Aldrich, Sigma Aldrich Inc., St. Louis, MO, USA) was used as the internal standard. Vials were topped with metal screw caps with 1.3 mm septa, blue silicone, and PTFE (Gerstel, Linthicum, MD, USA). Samples were analyzed in triplicate and the run order was randomized.

For instrument analysis the SPME fiber DVB/C-WR/PDMS, 50/30 µm, 10 mm (Restek, Restek Corporation, Bellefonte, PA, USA) was used. Samples were heated to 40 °C and agitated for 10 min at 250 rpm before sampling by the SPME fiber. The fiber was exposed to the sample for 30 min at 40 °C with an agitation speed of 250 rpm. The SPME fiber was desorbed in split mode with a 20:1 split ratio, the fiber was kept in the inlet for 10 min and the inlet temperature was held at 240 °C. The samples were analyzed using an 8890 gas chromatography system (Agilent, Agilent Technologies Inc., Santa Clara, CA, USA) as well as an MPS autosampler (Gerstel, Linthicum, MD, USA). A DB-Wax capillary column, 30 m, 0.250 mm, 0.25 µm (Agilent, Agilent Technologies Inc., Santa Clara, CA, USA) was used. Helium was used as the carrier gas at a constant flow or 1 mL/min. For analysis the oven parameters were as follows: 40 °C for 5 min, followed by an increase of 3 °C/min to 180 °C, then 30 °C/min to 240 °C where the temperature was held. The MSD interface was also held at 240 °C. Compounds were analyzed using deconvolution ranked by height and 50 retention time (RT) window size. Compounds were identified by the NIST 20 MS Search Version 2.4 using their respective qualifying ions shown in Supplemental Table S8.

#### 3.3.2. Stir Bar Sorptive Extraction

The stir-bar sorptive extraction (SBSE) method was based on a previously described procedure [76] with modifications for a DB-Wax capillary column. A volume of 5 mL sample was pipetted into 20 mL round-bottomed glass vials (Gerstel, Linthicum, MD, USA) containing 5 mL of ultra-pure water (Millipore, Sigma Aldrich Inc., St. Louis, MO, USA). 6.18 µL of 2-undecanone (Sigma Aldrich, Sigma Aldrich Inc., St. Louis, MO, USA) was used as the internal standard. All samples were twisted using a PDMS coated Twister^TM^ stir bar, 10 mm, 0.5 mm (Gerstel, Linthicum, MD, USA) for 1 h on a Twister^TM^ 20-position magnetic stirrer at 1000 rpm (Gerstel, Linthicum, MD, USA). Wine samples were analyzed in triplicate and the run order was randomized.

The initial thermal desorption unit (TDU) temperature was set at 40 °C then heated to 60 °C at a rate of 60 °C/min and a hold time of one minute for ramp 1. The TDU temperature was then raised to 280 °C at a rate of 270 °C/min and a 3-min hold time. Desorption mode was set at solvent venting for 1.33 min. The transfer temperature was 300 °C. Cyro cooling was utilized for 15 min, and the initial cooling injection system (CIS) temperature was −120 °C with an equilibration time of 0.20 min. The CIS temperature was raised to 280 °C at a rate of 12 °C/min and a hold time of 5 min. The samples were analyzed using an 8890 gas chromatography system (Agilent, Agilent Technologies Inc., Santa Clara, CA, USA) as well as a MPS autosampler (Gerstel, Linthicum, MD, USA). A DB-Wax capillary column, 30 m, 0.250 mm, 0.25 µm (Agilent, Agilent Technologies Inc., Santa Clara, CA, USA) was used. Helium was used as the carrier gas at a constant flow of 1.33 mL/min. For GC parameters, the inlet pressure was set at 15.63 psi with a septum purge flow of 3 mL/min. The mode was set at the solvent vent with a purge flow of 25.6 mL/min. Column flow rate was set at 1.28 mL/min with a pressure of 15.63 psi and a hold-up time of 1.82 min. The column oven started at 40 °C with a hold time of 1 min, followed by a ramp of 10 °C/min to 250 °C for a hold time of 3 min and a total run time of 25 min. The MSD interface was also held at 240 °C. Compounds were analyzed using deconvolution ranked by height and a 50 RT window size. Compounds were identified using NIST 20 MS Search Version 2.4 using their respective qualifying ions shown in Supplemental Table S8.

### 3.4. Descriptive Analysis and TCATA Sensory Panel

Members of the sensory panel (*n* = 8) were recruited by electronic mail (e-mail) and the project received California Polytechnic State University International Review Board (IRB) approval (IRB protocol #2020-058). The panel was composed of 50% females and 50% males with an age range of 21 to 60 years old. Panelists were screened for PROP sensitivity and salivary flow rate. Sensitivity to PROP was assessed using 6-*n*-propylthiouracil (PROP) (Fluka, Lot #: 1129436, Steinheim, Germany) following a previously described procedure [77]. The panel was composed of 50% super-tasters, 38% medium-tasters, and 13% non-tasters. The salivary flow rate collection procedure and calculation were performed as previously reported [41]. Additionally, panelists were screened for visual disorders and color perception deficiencies through pseudo-isochromatic color testing plates (Ishihara maps). None of the panelists showed color deficiencies based on this test.

#### 3.4.1. Panel Training

Wines were evaluated using general descriptive analysis (DA) and TCATA following previous procedures [8,40]. Panel training was conducted over seven sessions, each lasting from one hour to one and a half hours from August to September 2022. Descriptive analysis training focused on the evaluation procedure, attribute definition, standard review, and assessment of intensity. Nine attributes were selected by the panel which encompassed color and aroma. Attributes with standard compositions used during training are shown in Table 6. Initial training sessions focused on the identification of standards and the detection of the attributes in the research wines. The remaining training sessions incorporated the use of intensity assessment of attributes with a 15 cm, unstructured line scale. Finally, the last two training sessions had the panelists practice the use of the sensory software prior to formal evaluation. During training, the evaluation of color was conducted separately in clear, International Organization for Standardization (ISO) glasses while all other attributes were assessed using black glasses to mask the variation in color among treatments and avoid bias due to color.

**Figure 5:**
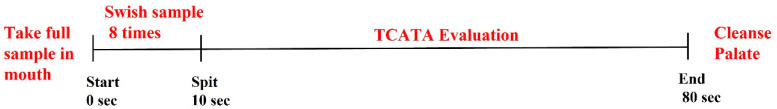
TCATA evaluation procedure.

TCATA training focused on the evaluation procedure, attribute definition, and retronasal aroma, taste, and mouthfeel standard review. The attribute list consisted of six categories selected by the panel. During training, panelists were asked to independently identify each standard followed by a group discussion where missed standards were reviewed again. For the panelists to understand the difference between retronasal aroma attributes and taste and mouthfeel attributes, standards were evaluated separately from each other. Taste and mouthfeel standards were evaluated using nose clips to block retronasal aromas. The TCATA evaluation procedure was practiced starting halfway through training. Panelists were instructed to take the full sample in their mouth and swish for 10 s. At the 10 s mark they were prompted to expectorate their sample and begin evaluation (Figure 5). Like TDS, the order of attributes was balanced across panelists [78]. A dual-stop feature was utilized when panelists no longer perceived any attributes before the 70 s was finished. The TCATA task lasted seventy seconds with results recorded at 0.5 s intervals by the software. Panelists were instructed to palate cleanse for fifteen seconds between samples.

#### 3.4.2. Formal Evaluations

Formal evaluations were conducted over five sessions in individual sensory cabinets at the J. Lohr Sensory Lab of the Wine & Viticulture department at California Polytechnic State University, San Luis Obispo, in September 2022. All replicates were evaluated three times. Descriptive analysis and TCATA were assessed in separate sessions. For descriptive analysis testing, 30 mL aliquots of samples were served. All samples regardless of the sensory method were served in clear, ISO glasses with four-digit random codes, and an aluminum foil top at room temperature. Wines were served monadically either under red lighting (Luna 3AO, 18:18W, Zaniboni Lighting, Clearwater, FL, USA) or for color assessment under daylight setting (Luna 3, 26:26W, Zaniboni Lighting, Clearwater, FL, USA). Order of serving was randomized according to William’s Latin Square Design. The assessment used a 15 cm line scale anchored at 1 cm and 14 cm with the terms “low” and “high” respectively.

To lower the bias of panelists when assessing the different modalities of the wine, color, and aroma were assessed separately. This resulted in six separate tests with 9 wines per test. The run order of the wines was randomized per test per session. Panelists assessed wines following the evaluation procedures described during training. Following each sample there was a forced palate cleansing of 15 s. A 10 min break was given halfway through each testing session.

TCATA formal evaluations were conducted over three sessions separate from DA and evaluated in individual sensory cabinets. All replicates were evaluated three times. 15 mL aliquots of sample were served at laboratory temperature (18 °C) in ISO glasses with four-digit random codes, and an aluminum foil top. Wines were served monadically under red lighting (Luna 3AO, 18:18W, Zaniboni Lighting, Clearwater, FL, USA). Order of serving was randomized according to William’s Latin Square Design. Panelists assessed eighteen wines per session, following the evaluation procedure described during training, with a 5 min break after nine samples. Following each sample there was a required palate cleansing of 15 s. Unsalted crackers (Nabisco unsalted tops, premium saltine crackers, East Hanover, NJ, USA) and water (Evian natural spring water, Evian, France) were provided for palate cleansing. All TCATA data collection was done in RedJade Sensory Software (RedJade, Silicon Valley, CA, USA). Panel performance was analyzed using the panel analysis feature for descriptive analysis and the TCATA function in the sensory package of XL STAT 2022.1.2 (Addinsoft, Paris, France) was utilized for agreement and repeatability during retronasal and mouthfeel assessment.

### 3.5. Statistical Analysis

The basic chemistry and phenolic data were analyzed by a one-way ANOVA (*p* < 0.05). Additionally, to understand the relationship between each main effect and the interaction in chemical and phenolic data, a two-way ANOVA was conducted (*p* < 0.05). Fisher’s Least Significant Differences (LSD) was used as the *post-hoc* comparison.

Quantification of the volatile compounds in wine samples was conducted by the internal standard method outlined in a previous procedure [75]. One-way and two-way ANOVAs were conducted on the SPME and SBSE data with Fisher’s LSD as the *post-hoc* test. To understand the potential relationship between volatile chemistry and sensory results, a Partial Least Square Regression was conducted whereby sensory attributes were the dependent variable and volatile compounds were the independent variable [79]. All statistical analyses were run in XLSTAT 2022.1.2 (Addinsoft, Paris, France).

Descriptive analysis data were analyzed by a one-way ANOVA (*p <* 0.05) on the wines. To understand the effect of each factor on the sensory responses, a two-way ANOVA (*p <* 0.05) was run on the descriptive analysis data. The *post-hoc* comparison for all statistical analyses was Fisher’s LSD test in XLSTAT 2022.1.2 (Addinsoft, Paris, France). Principal component analysis (PCA) using the correlation matrix with no rotation was applied to the wine sensory data, including the replicates, using R software (R Foundation for Statistical Computing, Vienna, Austria). Confidence ellipses indicating 95% confidence intervals were based on the multivariate distribution of Hotelling’s test for *p* < 0.05 and were constructed using the SensoMineR panellipse function on R [80].

TCATA curves were obtained using the tempR package [81] with the R-software version 4.2.2 (R Core Team, 2022). The tempR package follows a previously published protocol [10], by calculating the average citation proportions for each attribute (*y*-axis) over time (*x*-axis; 70 s) for each wine. The citation proportion of each sample and attributes at TCATA were recorded and grouped in contingency tables, where products and times were arranged in rows and attributes in columns. Data tables were submitted to Principal Component Analysis (PCA) using the approach described [82] but with attribute standardization. Trajectories were smoothed along each dimension independently and PCA biplots were visualized using the tempR package [81]. Selected time parameters (Table 7) were extracted from TCATA curves and analyzed by attribute using a one-way ANOVA (*p* < 0.05) and Fisher’s LSD test in XLSTAT 2022.1.2 (Addinsoft, Paris, France).

Panelist salivary flow rate data were analyzed by a one-way ANOVA (*p* < 0.05) to determine the spread of variance in the panel. These values were converted to categorical data by designating values above the overall mean as a high flow rate and below the mean as a low flow rate [41]. A separate one-way ANOVA (*p* < 0.05) was run on the categorical flow rates to compare these values with the perception of the astringency sub-qualities suede and velvet as well as the fruit aroma. Statistical analyses were conducted in XLSTAT 2022.1.2 (Addinsoft, Paris, France).

## 4. Conclusions

Based on the TCATA assessment, the most optimal treatment of this experiment was the Cold/Hot PD wines as the wines had a significantly higher citation proportion of fruit flavor and velvet astringency perception relative to the other winemaking treatments. These two terms bear potentially positive hedonic connotations for consumers. Therefore, Grenache winemaking may benefit from a combination of contrasting fermentation temperatures during maceration. For instance, the practice of controlled cold soak at low temperatures, ideally inoculated with a selected yeast strain, followed by a relatively warm to hot alcoholic fermentation temperature, could lead to results comparable with those of the present study. Generally, gentler cap management regimes that minimize overexposure to oxygen may lead to more aromatic retention in Grenache wines. However, this minimalist intervention, if applied, will have to be carefully managed to avoid or minimize the production of reduction aromas.

In summary, for Grenache wines, it is recommended to utilize a Cold/Hot fermentation temperature as it will lead to more retronasal and mouthfeel and a balance of fruity and reduced aromas. Both fermentation temperature and cap management appear to be variety-specific, thus further research on both these factors on popular wine varietals needs to be carried out. Finally, the present study represents a contribution with the main volatile compounds (e.g., β-damascenone and esters in the Cold fermented wines; hexanol in PD wines, which may be potentially responsible for a hot aroma and mouthfeel), and sensory characteristics (red fruit, tropical fruit, white pepper, and rose) of Grenache wines grown in the Mediterranean climate of the Central Coast of California.

## Figures and Tables

**Table 1:** One-way analysis of variance (ANOVA) showing mean separation and *p*-values of descriptive sensory attributes of Grenache wines assessed by a trained panel (*n* = 8).

Treatments	Saturation	Purple Hue	Overall Aroma Intensity	Reduction	Rose	Red Fruit	Tropical Fruit	White Pepper	Mushroom	Hot
Cold PD	3.49 c ^1^	5.02 c	7.61 ab	3.53 bc	5.67 a	5.57 a	5.06 ab	3.23 ab	2.70 ab	6.55 a
Cold No PD	2.42 d	4.36 c	7.81 ab	5.10 a	4.58 b	5.03 a	4.42 b	3.30 ab	2.99 ab	6.07 a
Cold/Hot PD	6.29 b	7.00 b	7.99 a	4.20 ab	4.83 b	5.41 a	4.96 ab	3.49 a	2.84 ab	6.94 a
Cold/Hot No PD	6.65 b	6.84 b	8.03 a	4.88 a	4.95 ab	5.44 a	5.26 a	3.51 a	3.09 a	6.10 a
Hot PD	6.53 b	8.21 a	7.17 b	2.77 c	5.05 ab	5.71 a	4.72 ab	2.80 b	2.35 b	6.90 a
Hot No PD	7.98 a	8.67 a	7.83 ab	5.08 a	4.54 b	5.43 a	4.88 ab	3.22 ab	3.30 a	6.25 a
*p*-value ^2^	**<0.0001**	**<0.0001**	0.291	**<0.0001**	0.058	0.744	0.386	0.326	0.144	0.261

^1^ Different letters within columns indicate a significant difference for Fisher’s least significant differences test (*p <* 0.05). ^2^ Significant *p*-values are shown in bold fonts.

**Table 2:** One-way analysis of variance (ANOVA) of the phenolic composition of Grenache wines. Values represent the mean of three replicates followed by the standard error of the mean (*n* = 3).

Treatment	Anthocyanins (mg/L Malvidin-3-Glucoside)	SPP	LPP	TPP	Total Tannins (mg/L CE)	Total Phenolics (mg/L CE)
Cold PD	187 ± 9.23 b ^1^	0.480 ± 0.03 c	0.026 ± 0.02 bc	0.505 ± 0.05 c	26.4 ± 3.70 b	487 ± 36.4 b
Cold No PD	178 ± 6.98 b	0.432 ± 0.01 c	0.004 ± 0.01 c	0.436 ± 0.01 c	22.8 ± 4.11 b	509 ± 17.1 b
Cold/Hot PD	280 ± 4.61 a	0.656 ± 0.02 b	0.070 ± 0.02 bc	0.727 ± 0.01 b	94.8 ± 7.55 a	800 ± 19.4 a
Cold/Hot No PD	309 ± 8.27 a	0.680 ± 0.01 b	0.022 ± 0.02 bc	0.702 ± 0.03 b	87.7 ± 14.7 a	862 ± 44.4 a
Hot PD	220 ± 5.39 b	0.718 ± 0.02 b	0.209 ± 0.01 a	0.927 ± 0.02 a	96.8 ± 13.0 a	735 ± 19.6 a
Hot No PD	299 ± 41.2 a	0.793 ± 0.04 a	0.085 ± 0.04 b	0.878 ± 0.07 a	81.0 ± 18.8 a	893 ± 112 a
*p*-value ^2^	**0.001**	**<0.0001**	**0.001**	**<0.0001**	**0.001**	**0.000**

^1^ Different letters within columns indicate a significant difference for Fisher’s least significant differences test (*p* < 0.05). ^2^ Significant *p*-values are shown in bold fonts. SPP: Small polymeric pigments; LPP: Large polymeric pigments; TPP: total polymeric pigments; CE: catechin equivalents.

**Table 3:** One-way analysis of variance (ANOVA) of the concentration of volatile compounds (µg/L) in Grenache wines. The average odor activity value (OAV) for each wine is shown in parentheses for each volatile compound. Values represent the mean of three replicates (*n* = 3).

Compounds	Cold PD	Cold No PD	Cold/Hot PD	Cold/Hot No PD	Hot PD	Hot No PD	*p*-Value	Source ^1^
Esters								
Isobutyl acetate	n.d. ^2^ (0.00)	34.1 (0.016) bc ^3^	n.d. (0.00)	n.d. (0.00)	69.5 (0.033) b	141 (0.067) a	**0.002 ^4^**	[52]
Ethyl butyrate	176 (3.42) bc	156 (3.02) cd	197 (3.83) ab	229 (4.46) a	137 (2.67) d	191 (3.71) bc	**0.002**	[53]
Hexyl acetate	6.44 (2.22) a	2.77 (0.955) b	4.79 (1.65) ab	5.20 (1.79) a	n.d. (0.00)	n.d. (0.00)	**0.000**	[54]
Isoamyl acetate	727 (19.7) cd	752 (20.3) cd	846 (22.9) bc	1138 (30.7) a	611 (16.5) d	998 (27.0) ab	**0.001**	[53]
Ethyl hexanoate	631 (3.16) a	523 (2.62) bc	613 (3.06) ab	687 (3.44) a	440 (2.20) c	490 (2.45) c	**0.002**	[55]
Ethyl lactate	22,125 (0.143) a	26,695 (0.173) a	26,704 (0.173) a	33,023 (0.214) a	35,740 (0.231) a	26,006 (0.168) a	0.365	[56]
Ethyl heptanoate	0.994 (-) a ^5^	n.d. (-)	0.464 (-) a	n.d. (-)	0.855 (-) a	n.d. (-)	0.165	- ^5^
Ethyl octanoate	356 (2.21) b	289 (1.80) b	418 (2.60) ab	525 (3.26) a	336 (2.09) b	294 (1.83) b	**0.021**	[53]
Ethyl decanoate	10.7 (0.010) a	8.33 (0.007) a	10.8 (0.010) a	12.3 (0.011) a	9.49 (0.009) a	5.49 (0.005) a	0.771	[57]
Diethyl succinate	337 (0.002) b	316 (0.002) b	354 (0.002) b	419 (0.002) b	892 (0.004) a	546 (0.003) ab	**0.043**	[56]
Ethyl hexadecanoate	396 (-) ab	614 (-) ab	163 (-) b	439 (-) ab	871 (-) a	520 (-) ab	0.234	-
Phenylethyl acetate	8.15 (0.033) bc	11.0 (0.044) abc	4.65 (0.019) c	16.7 (0.067) a	8.20 (0.033) bc	14.3 (0.057) ab	0.081	[57]
Ethyl cinnamate	1.12 (1.02) b	1.89 (1.72) ab	1.49 (1.35) ab	1.98 (1.80) ab	2.01 (1.82) ab	2.27 (2.07) a	0.142	[58]
Total Esters	24,776 a	29,403 a	29,317 a	36,497 a	39,118 a	29,209 a	0.340	
Nor-isoprenoids								
β-damascenone	0.757 (15.1) a	0.680 (13.6) a	n.d. (0.00)	n.d. (0.00)	n.d. (0.00)	n.d. (0.00)	**0.050**	[59]
Terpenes								
Citronellol	15.1 (0.838) a	14.5 (0.805) a	11.5 (0.636) b	11.6 (0.647) b	13.0 (0.723) ab	14.6 (0.809) a	0.057	[59]
Alcohols								
1-hexanol	2293 (2866) a	1462 (1828) b	2550 (3188) a	1664 (2080) b	2519 (3145) a	1592 (1990) b	**0.000**	[59]
1-octanol	n.d. (0.00)	n.d. (0.00)	1.74 (0.016) a	n.d. (0.00)	1.98 (0.018) a	n.d. (0.00)	0.570	[60]
1-nonanol	0.972 a (-)	n.d. (-)	0.677 a (-)	0.916 a (-)	n.d. (-)	n.d. (-)	0.692	-
Isobutanol	7982 (0.200) bcd	11,602 (0.290) abc	7710 (0.193) cd	7013 (0.175) d	12,682 (0.317) a	12,451 (0.311) ab	**0.047**	[56]
Isoamyl alcohol	49,280 (1.64) a	50,106 (1.67) a	40,457 (1.35) a	55,908 (1.86) a	71,490 (2.38) a	44,475 (1.48) a	0.494	[58]
Phenylethyl alcohol	15,660 (1.12) de	15,223 (1.09) e	19,369 (1.38) cd	20,646 (1.47) c	25,698 (1.84) b	32,479 (2.32) a	**<0.0001**	[58]
Total Alcohols	75,216 ab	78,395 ab	70,088 b	85,230 ab	112,387 a	90,997 ab	0.251	
Aldehydes								
Benzaldehyde	20.0 (0.001) a	18.0 (0.001) a	13.6 (0.001) a	32.2 (0.002) a	12.4 (0.001) a	n.d. (0.00)	0.767	[58]

^1^ Source of detection threshold used to calculate OAV. ^2^ Volatile compound not detected during analysis. ^3^ Different letters within columns indicate a significant difference for Fisher’s least significant differences test (*p* < 0.05). ^4^ Significant *p*-values are shown in bold fonts. ^5^ (-) indicates that there was no literature detection threshold found.

**Table 4:** One-way analysis of variance (ANOVA) of the basic chemical composition of Grenache wines. Values represent the mean of three replicates followed by the standard error of the mean (*n* = 3).

Treatments	Ethanol (*v*/*v*%)	pH	Titratable Acidity (g/L)	Acetic Acid (g/L)	Glucose + Fructose (g/L)	Lactic Acid (g/L)	Malic Acid (g/L)
Cold PD	13.3 ± 0.17 a ^1^	3.61 ± 0.04 ab	5.52 ± 0.12 d	0.267 ± 0.04 b	0.140 ± 0.03 ab	1.24 ± 0.03 ab	0.060 ± 0.01 a
Cold No PD	13.1 ± 0.23 ab	3.69 ± 0.01 a	5.67 ± 0.06 cd	0.380 ± 0.03 a	0.127 ± 0.01 ab	1.18 ± 0.02 ab	0.060 ± 0.01 a
Cold/Hot PD	13.1 ± 0.11 ab	3.54 ± 0.01 c	5.74 ± 0.04 bcd	0.180 ± 0.01 c	0.113 ± 0.02 ab	1.25 ± 0.01 a	0.050 ± 0.01 a
Cold/Hot No PD	12.8 ± 0.04 b	3.55 ± 0.01 bc	5.97 ± 0.02 ab	0.177 ± 0.01 c	0.150 ± 0.01 a	1.26 ± 0.01 a	0.040 ± 0.01 a
Hot PD	13.1 ± 0.16 ab	3.53 ± 0.02 c	5.87 ± 0.06 bc	0.287 ± 0.02 b	0.097 ± 0.01 b	1.15 ± 0.03 bc	0.043 ± 0.01 a
Hot No PD	12.8 ± 0.17 ab	3.62 ± 0.03 ab	6.19 ± 0.12 a	0.277 ± 0.03 b	0.120 ± 0.01 ab	1.09 ± 0.05 c	0.047 ± 0.01 a
*p*-value ^2^	0.300	**0.004**	**0.001**	**0.001**	0.194	**0.011**	0.318

^1^ Different letters within columns indicate a significant difference for Fisher’s least significant differences test (*p* < 0.05). ^2^ Significant *p*-values are shown in bold fonts.

**Table 5:** One-way analysis of variance (ANOVA) of sensory attributes and time extracted from TCATA curves parameters evaluated by the trained panel (*n* = 8).

Attribute	AUC ^1^	T_max_ (Seconds)	V_max_ (Citation Proportion)	T_first_ (Seconds)	T_last_ (Seconds)	Length (Seconds)
Acidity	24.2 b ^2^	24.1 d	0.875 a	14.8 d	69.4 b	54.7 bc
Bitter	28.6 a	28.4 bc	0.861 ab	15.3 cd	76.7 a	61.4 a
Fruit	19.4 c	23.9 d	0.781 c	14.6 d	66.1 b	51.5 c
Hot	14.5 d	22.4 d	0.778 c	15.2 d	53.1 d	37.9 d
Reduction	15.3 d	25.2 cd	0.660 d	16.1 c	58.6 c	42.5 d
Suede	28.9 a	30.1 ab	0.785 bc	17.6 b	78.9 a	61.3 a
Velvet	19.2 c	32.9 a	0.576 e	18.6 a	75.8 a	57.2 ab
*p*-value ^3^	**<0.0001**	**<0.0001**	**<0.0001**	**<0.0001**	**<0.0001**	**<0.0001**

^1^ AUC: total intensity response; T_max_: time of maximum citation proportion; V_max_: maximum citation proportion; T_first:_ time of first citation proportion per attribute; T_last_: time of last citation proportion per attribute; Length: total time attribute was perceived. ^2^ Different letters within columns indicate a significant difference for Fisher’s least significant differences test, *p* < 0.05. ^3^ Significant *p*-values are shown in bold fonts.

**Table 6:** Color, aroma, taste, flavor, and mouthfeel attribute standard composition.

Attribute		Standard Composition
Color	Purple Hue	High: L* = 39.93, C* = 61.92, a* = 60.82, b* = 11.63 ^1^
		Low: L* = 86.40, C* = 15.57, a* = 15.13, b* = 3.66 ^2^
	Saturation	High: L* = 39.93, C* = 61.92, h = 10.83 ^1^
		Low: L* = 86.40, C* = 15.57, h = 13.60 ^2^
Aroma ^3^	Reduction	100 mL of base reduction solution; 116.94 g raw white onion (Signature Farms, Better Living Brands LLC, Pleasanton, CA, USA), 112.02 g cooked, white onion (Signature Farms, Better Living Brands LLC, Pleasanton, CA, USA) on medium until browned and soaked in 1000 mL of base wine overnight.
	Rose	80 mL rose water (Fee Brothers, Fee Brothers, Rochester, NY, USA), 99.2 mL rose syrup (Monin, Monin Inc., Clearwater, FL, USA)
	Red Fruit	51.54 g mashed, fresh raspberries (Fresh Kampo, Meridian Fruits, Periban Los Reyes, SN 60440, Mexico), 89.08 g cut, fresh strawberries (Signature Farms, Better Living Brands LLC, Pleasanton, CA, USA)
	Tropical Fruit	23.62 g crème de banana syrup (Torani, R. Torre & Company, San Leandro, CA, USA), 12.98 g mango syrup (Torani, R. Torre & Company, San Leandro, CA, USA)
	White Pepper	6.60 g white pepper (First Street, Amerifoods Trading Co, Los Angeles, CA, USA)
	Mushroom	52.34 g chopped, raw Baby Bello mushrooms (Signature Farms, Better Living Brands LLC, Pleasanton, CA, USA)
	Hot	444 mL of vodka (New Amsterdam, New Amsterdam Spirits Company, Modesto, CA, USA)
Retronasal Aromas	Reduction	100 mL of base reduction solution; 116.94 g raw white onion (Signature Farms, Better Living Brands LLC, Pleasanton, CA, USA), 112.02 g cooked, white onion (Signature Farms, Better Living Brands LLC, Pleasanton, CA, USA) on medium until browned and soaked in 1000 mL of base wine overnight.
	Fruit	61.26 g cut, fresh strawberries (Signature Farms, Better Living Brands LLC, Pleasanton, CA, USA), 13.26 g strawberry preserves (Bonne Maman, Andros, France), 16.31 g wild blueberry preserves (Bonne Maman, Andros, France), 18.39 g cherry preserves (Bonne Maman, Andros, France), 13.95 g blackberry syrup (Torani, R. Torre & Company, San Leandro, CA, USA), 15.29 g cherry syrup (Torani, R. Torre & Company, San Leandro, CA, USA)
	Hot	444 mL of vodka (New Amsterdam, New Amsterdam Spirits Company, Modesto, CA, USA)
Taste	Acidity ^4^	1.23 g tartaric acid (LD Carlson Company, LD Carlson Company, Kent, OH, USA)
	Bitter ^4^	1.32 g caffeine, anhydrous (Sigma Aldrich, 1003363509, Sigma-Aldrich Co., St. Louis, MO, USA)
Mouthfeel	Suede ^5^	Suede dress (Willow Ridge)
	Velvet ^5^	Velvet jacket (Chico’s)

^1^ 2021 Graciano research wine, CIE Lab Measurements. ^2^ 2021 Graciano research and 50 mL of water, CIE Lab Measurements. ^3^ Prepared to 1000 mL of Franzia Merlot (Franzia, Franzia Vineyards, Ripon, CA, USA). ^4^ Prepared in 750 mL of Franzia Merlot (Franzia, Franzia Vineyards, Ripon, CA, USA). ^5^ Cut two 5.10 cm × 12.7 cm pieces. One was glued to the canvas board; another was pulled through the canvas hole and allowed to dangle.

**Table 7:** Definitions of time parameters extracted from TCATA curves.

Time Parameter	Definition
V_max_	Maximum citation proportion
T_max_	Time of maximum citation proportion
Area Under Curve	Total intensity response
T_first_	Time of first citation proportion per attribute
T_last_	Time of last citation proportion per attribute
Length	Total time attribute was perceived (T_first_–T_last_)

## Data Availability

Not applicable.

## Supplementary Materials

The following supporting information can be downloaded at: https://www.mdpi.com/article/10.3390/molecules28104230/s1, Table S1: Two-way analysis of variance (ANOVA) for the main effects fermentation temperature and cap management with interaction showing the mean separation and *p*-values of descriptive sensory attributes of Grenache wines assessed by a trained panel (*n* = 8); Table S2: Two-way analysis of variance (ANOVA) for the main effects of fermentation temperature and cap management with interaction showing the mean separation and *p*-values of the phenolic composition of Grenache wines. Values represent the mean of three replicates followed by the standard error of the mean (*n* = 3); Table S3: Two-way analysis of variance (ANOVA) for the main effects of fermentation temperature and cap management with interaction showing the mean separation and *p*-values for the concentration of volatile compounds (µg/L) in Grenache wines. Values represent the mean of three replicates (*n* = 3); Table S4: Two-way analysis of variance (ANOVA) for the main effects of fermentation temperature and cap management with interaction showing the mean separation and *p*-values of the basic chemical composition of Grenache wines. Values represent the mean of three replicates followed by the standard error of the mean (*n* = 3); Table S5: One-way analysis of variance (ANOVA) of the saliva (mL/min) for each panelist. Values represent the mean of three salivary flow rate replicates followed by the standard error of the mean (*n* = 3); Table S6: One-way analysis of variance (ANOVA) of time to onset of perception for astringency subqualities (suede, velvet) and retronasal fruit compared to salivary flow rate (mL/min); Table S7: Compounds evaluated using SPME and SBSE on the GC-MS, CAS numbers, manufacturers, and purity. Table S8: Selected volatile compounds for stir bar sorptive extraction (SBSE) and solid-phase microextraction (SPME) analysis with a concentration in wine according to the literature (µg/L), volume added to the wine (µL), and concentration in model wine (µg/L). References [83,84,85,86,87,88,89,90,91,92,93] are cited in the supplementary materials.
