# Supplementary material for: The Impact of Fermentation Temperature and Cap Management on Selected Volatile Compounds and Temporal Sensory Characteristics of Grenache Wines from the Central Coast of California
Source: Molecules. 2023 May 22;28(10):4230. doi: 10.3390/molecules28104230 (PMC10224402; doi:10.3390/molecules28104230)
Supplement: Supplementary file 1 [file molecules-28-04230-s001.zip › molecules-2411648-supplementary.pdf]

Supplemental Table S1. Two-way analysis of variance (ANOVA) for the main effects fermentation temperature and cap management with interaction showing the mean separation and *p*-values of descriptive sensory attributes of Grenache wines assessed by a trained panel (n = 8).

	**Saturation**	**Purple hue**	**Overall Aroma Intensity**	**Reduction**	**Rose**	**Red fruit**	**Tropical fruit**	**White pepper**	**Mushroom**	**Hot**
					**Fermentation Temperature**					
Cold	2.95 c [1]	4.69 c	7.71 a	4.31 a	5.12 a	5.30 a	4.74 a	3.26 ab	2.84 a	6.31 a
Cold/Hot	6.47 b	6.92 b	8.01 a	4.54 a	4.89 a	5.43 a	5.11 a	3.50 a	2.97 a	6.52 a
Hot	7.25 a	8.44 a	7.50 a	3.92 a	4.80 a	5.57 a	4.80 a	3.01 b	2.82 a	6.57 a
***p*-value[2]**	**<0.0001**	**<0.0001**	0.199	0.208	0.488	0.682	0.372	0.123	0.836	0.724
					**Cap Management**					
PD	5.44 a	6.74 a	7.59 a	3.50 b	5.18 a	5.56 a	4.91 a	3.17 a	2.63 b	6.80 a
No PD	5.68 a	6.62 a	7.89 a	5.02 a	4.69 b	5.39 a	4.85 a	3.34 a	3.13 a	6.14 b
***p*-value**	0.358	0.707	0.195	**<0.0001**	**0.031**	0.295	0.795	0.376	**0.019**	**0.018**
					**Fermentation Temperature × Cap Management**					
***p*-value**	**<0.0001**	**<0.0001**	0.291	**<0.0001**	0.058	0.744	0.386	0.326	0.144	0.261

[1]Different letters within columns indicate a significant difference for Fisher's least significant difference test ($p < 0.05$).

[2]Significant *p*-values are shown in bold fonts.

Supplemental Table S2. Two-way analysis of variance (ANOVA) for the main effects fermentation temperature and cap management with interaction showing the mean separation and *p*-values of the phenolic composition of Grenache wines. Values represent the mean of three replicates followed by the standard error of the mean (n = 3).

	**Anthocyanins (mg/L malvidin-3-glucoside)**	**SPP**	**LPP**	**TPP**	**Total Tannins (mg/L CE)**	**Total Phenolics (mg/L CE)**
			**Fermentation Temperature**			
Cold	182 ± 5.57 b[1]	0.456 ± 0.02 c	0.015 ± 0.01 c	0.471 ± 0.03 c	24.6 ± 2.61 b	500 ± 18.6 b
Cold/Hot	294 ± 7.70 a	0.668 ± 0.01 b	0.046 ± 0.02 b	0.714 ± 0.02 b	91.3 ± 7.56 a	831 ± 25.7 a
Hot	260 ± 25.6 a	0.756 ± 0.03 a	0.147 ± 0.03 a	0.903 ± 0.03 a	88.9 ± 10.8 a	814 ± 62.1 a
***p*-value[2]**	**0.001**	**<0.0001**	**0.004**	**<0.0001**	**<0.0001**	**<0.0001**
			**Cap Management**			
PD	229 ± 14.0 a	0.618 ± 0.04 a	0.102 ± 0.03 a	0.720 ± 0.06 a	72.7 ± 12.4 a	674 ± 49.5 a
No PD	262 ± 24.4 a	0.635 ± 0.05 a	0.037 ± 0.02 a	0.672 ± 0.07 a	63.8 ± 12.5 a	754 ± 71.0 a
***p*-value**	0.260	0.801	0.085	0.615	0.620	0.366
			**Fermentation Temperature × Cap Management**			
***p*-value**	**0.001**	**<0.0001**	**0.001**	**<0.0001**	**0.001**	**0.000**

[1]Different letters within columns indicate a significant difference for Fisher's least significant difference test ($p < 0.05$).

[2]Significant *p*-values are shown in bold fonts.

Supplemental Table S3. Two-way analysis of variance (ANOVA) for the main effects fermentation temperature and cap management with interaction showing the mean separation and *p*-values for the concentration of volatile compounds (µg/L) in Grenache wines. Values represent the mean of three replicates (n=3).

**Compounds**	**Temperature**				**Cap Management**			**Temperature x Cap Management**
	**Cold**	**Cold/Hot**	**Hot**	***p*-value**	**PD**	**No PD**	***p*-value**	***p*-value**
Esters								
Isobutyl acetate	17.0 b[1]	n.d.[2]	105 a	**0.001**[3]	23.2 a	58.5 a	0.227	**0.002**
Ethyl butyrate	166 b	213 a	164 b	**0.012**	170 a	192 a	0.194	**0.002**
Hexyl acetate	4.60 a	4.99 a	n.d.	**0.000**	3.74 a	2.66 a	0.426	**0.000**
Isoamyl acetate	739 b	992 a	805 ab	0.073	728 b	963 a	**0.009**	**0.001**
Ethyl hexanoate	577 a	650 a	465 b	**0.001**	561 a	567 a	0.910	**0.002**
Ethyl lactate	24,410 a	29,864 a	30,873 a	0.358	28,190 a	28,575 a	0.924	0.365
Ethyl heptanoate	0.497 a	0.232 a	0.427 a	0.783	0.771 a	n.d.	**0.006**	0.165
Ethyl octanoate	322 b	472 a	315 b	**0.006**	370 a	370 a	0.995	**0.021**
Ethyl decanoate	9.50 a	11.5 a	7.49 a	0.453	10.3 a	8.70 a	0.539	0.771
Diethyl succinate	327 b	387 b	719 a	**0.015**	528 a	427 a	0.448	**0.043**
Ethyl hexadecanoate	505 a	301 a	696 a	0.150	477 a	524 a	0.785	0.234
Phenylethyl acetate	9.56 a	10.7 a	11.3 a	0.887	7.00 b	14.0 a	**0.006**	0.081
Ethyl cinnamate	1.51 a	1.73 a	2.14 a	0.158	1.54 a	2.05 a	0.057	0.142
Total Esters	27,089 a	32,907 a	34,163 a	0.328	31,070 a	31,703 a	0.881	0.340
Nor-isoprenoids								
β-damascenone	0.719 a	n.d.	n.d.	**0.002**	0.252 a	0.227 a	0.912	**0.050**
Terpenes								
Citronellol	14.8 a	11.5 b	13.8 a	**0.006**	13.2 a	13.6 a	0.697	0.057
Alcohols								
1-hexanol	1878 a	2107 a	2054 a	0.743	2453 a	1573 b	**<0.0001**	**0.000**
1-octanol	n.d.	0.870 a	0.988 a	0.614	1.24 a	n.d.	0.151	0.570
1-nonanol	0.486 a	0.796 a	n.d.	0.402	0.550 a	0.305 a	0.618	0.692
Isobutanol	9,792 ab	7,361 b	12,566 a	**0.011**	9,458 a	10,356 a	0.573	**0.047**
Isoamyl alcohol	49,693 a	48,182 a	57,983 a	0.669	53,742 a	50,163 a	0.708	0.494
Phenylethyl alcohol	15,442 c	20,007 b	29,088 a	**<0.0001**	20,242 a	22,783 a	0.421	**<0.0001**
Total Alcohols	76,805. a	77,659 a	101,692 a	0.093	85,897 a	84,874 a	0.927	0.251
Aldehydes								
Benzaldehyde	19.0 a	22.9 a	6.19 a	0.471	15.3 a	16.7 a	0.905	0.767

[1]Different letters within columns indicate a significant difference for Fisher's least significant differences test ($p < 0.05$).
[2]Compound not detected during analysis.
[3]Significant *p*-values are shown in bold fonts.

Supplemental Table S4. Two-way analysis of variance (ANOVA) for the main effects fermentation temperature and cap management with interaction showing the mean separation and *p*-values of basic chemical composition of Grenache wines. Values represent the mean of three replicates followed by the standard error of the mean (n = 3).

	Ethanol (v/v%)	pH	Titratable acidity (g/L)	Acetic acid (g/L)	Glucose + Fructose (g/L)	Lactic acid (g/L)	Malic acid (g/L)
				**Fermentation Temperature**			
Cold	13.2 ± 0.13 a[1]	3.65 ± 0.03 a	5.59 ± 0.07 b	0.323 ± 0.03 a	0.133 ± 0.01 a	1.21 ± 0.02 a	0.060 ± 0.01 a
Cold/Hot	12.9 ± 0.09 a	3.54 ± 0.01 b	5.85 ± 0.05 a	0.178 ± 0.01 b	0.132 ± 0.01 a	1.25 ± 0.01 a	0.045 ± 0.01 b
Hot	13.0 ± 0.12 a	3.57 ± 0.03 b	6.03 ± 0.09 a	0.282 ± 0.01 a	0.108 ± 0.01 a	1.12 ± 0.03 b	0.045 ± 0.01 b
***p*-value[2]**	0.306	**0.009**	**0.003**	**0.001**	0.221	**0.002**	0.069
				**Cap Management**			
PD	13.1 ± 0.08 a	3.56 ± 0.02 a	5.71 ± 0.07 b	0.244 ± 0.02 a	0.117 ± 0.01 a	1.21 ± 0.02 a	0.051 ± 0.01 a
No PD	12.9 ± 0.09 a	3.62 ± 0.02 a	5.94 ± 0.08 a	0.278 ± 0.03 a	0.132 ± 0.01 a	1.17 ± 0.03 a	0.049 ± 0.01 a
***p*-value**	0.057	0.073	**0.046**	0.393	0.241	0.305	0.734
				**Fermentation Temperature × Cap Management**			
***p*-value**	0.300	**0.004**	**0.001**	**0.001**	0.194	**0.011**	0.318

[1]Different letters within columns indicate a significant difference for Fisher's least significant difference test ($p < 0.05$).

[2]Significant *p* values are shown in bold fonts.

Supplemental Table S5. One-way analysis of variance (ANOVA) of the saliva (mL/min) for each panelist. Values represent the mean of three salivary flow rate replicates followed by the standard error of the mean (n = 3).

Panelist Code	Salivary flow rate (mL/min)
325	3.18 ± 0.19 a[1]
193	3.02 ± 0.04 ab
244	2.88 ± 0.11 abc
368	2.83 ± 0.10 abc
715	2.71 ± 0.06 bc
837	2.53 ± 0.10 c
783	1.58 ± 0.29 d
442	1.39 ± 0.11 d
p-value[2]	**<0.0001**

[1]Different letters within the same column indicate a significant difference for Fisher's least significant difference test ($p < 0.05$).

[2]Significant *p*-values are shown in bold fonts.

Supplemental Table S6. One-way analysis of variance (ANOVA) of time to onset of perception for astringency subqualities (suede, velvet) and retronasal fruit compared to salivary flow rate (mL/min).

Salivary Flow Rate (mL/min)	Time of first suede perception (sec)	Time of first velvet perception (sec)	Time of fruit perception (sec)
LF	12.1 a[1]	5.05 a	6.88 a
HF	8.70 b	4.76 a	6.33 a
p-value[2]	**0.001**	0.749	0.500

[1]Different letters within columns indicate a significant difference for Fisher's least significant difference test, $p < 0.05$.
[2]Significant *p*-values are shown in bold fonts.

Supplemental Table S7. Compounds evaluated using SPME and SBSE on the GC-MS, CAS numbers, manufacturers, and purity.

Compound	CAS Number	Manufacturer	Purity
		**Esters**	
Isobutyl acetate	110-19-0	TCI	99%
Ethyl isobutyrate	97-62-1	Alfa Aesar	98%
Ethyl butyrate	105-54-5	Alfa Aesar	99%
Ethyl propionate	105-37-3	TCI	99%
Isoamyl acetate	123-92-2	Acros Organics	99%
Hexyl acetate	142-92-7	Sigma Aldrich	98.5%
Ethyl hexanoate	123-66-0	TCI	99%
Ethyl heptanoate	106-30-9	Sigma Aldrich	99%
Ethyl octanoate	106-32-1	TCI	98%
Ethyl decanoate	110-38-3	TCI	98%
Diethyl succinate	123-25-1	Sigma Aldrich	99.5%
Ethyl hexadecanoate	56219-10-4	TCI	95%
Phenylethyl acetate	103-45-7	Sigma Aldrich	99%
Ethyl cinnamate	4192-77-2	TCI	99%
Ethyl lactate	97-64-3	Spectrum	98%
		**Nor-isoprenoids**	
β-damascenone	23726-91-2	Sigma Aldrich	90%
β-ionone	14901-07-6	Sigma Aldrich	96%
		**Terpenes**	
Citronellol	7540-51-4	Sigma Aldrich	95%
trans-farnesol	4602-84-0	Sigma Aldrich	96%
		**Alcohols**	
1-hexanol	111-27-3	Sigma Aldrich	99%
1-octanol	111-87-5	Sigma Aldrich	99%
1-nonanol	143-08-8	Sigma Aldrich	98%
Isoamyl alcohol	123-51-3	Sigma Aldrich	95%
Isobutanol	78-83-1	Fischer Chemical	95%
Phenylethyl alcohol	98-85-1	Acros Organics	98%
		**Aldehydes**	
Benzaldehyde	10-52-7	Sigma Aldrich	99%
		**Sulfur Compounds**	
Methionol	505-10-2	Sigma Aldrich	98%
		**Internal Standard**	
2-undecanone	112-12-9	Sigma Aldrich	98%

Supplemental Table S8. Selected volatile compounds for stir bar sorptive extraction (SBSE) and solid-phase microextraction (SPME) analysis with concentration in wine according to the literature (μg/L), volume added to wine (μL), and concentration in model wine (μg/L).

Stock Solution	Volatile Compound	Typical Concentration in Wine (μg/L)	Source	Volume added to model wine (μL)	Concentration in model wine (μg/L)	Qualifying ions (m/z)
1	Trans-farnesol[1]	0.7 - 2.4	[83]	2	1.7	69,81,107
2	Ethyl heptanoate	5.50	[64]	5	4.63	70,88,113
	1-nonanol	9.01	[84]	5	4.05	56,83
	1-octanol	10	[64]	5	4.09	70, 84
3	Hexyl acetate	200	[85]	140	121	56,69
4	Ethyl isobutryrate[1]	200	[86]	200	170	71,88,116
	β-ionone[1]	240	[84]	200	181	91,135,177
	Methionol[1]	3.70 -207	[87]	200	196	97, 126
5	β-damascenone	425	[88]	450	378	123, 192
	Ethyl propionate[1]	301- 489	[87]	450	396	57,75, 102
	Diethyl succinate	500	[89]	450	468	73, 101, 129
6	Benzaldehyde	5 - 660	[90]	600	618	51, 77,106
	Isobutyl acetate	665	[88]	600	517	73, 86
7	Ethyl cinnamate	825	[88]	750	780	103, 176
	Ethyl decanoate	931	[88]	750	632	101, 115, 155
8	Ethyl butyrate	1,456	[88]	1,000	870	68, 71,88
	2-phenylethyl acetate	1,500	[88]	1,000	991	104, 91,78
	β-citronellol	1,563	[88]	1,000	843	69, 81,95
	Isoamyl acetate	500 -1,619	[88]	1,000	862	70, 87, 55
9	Phenylethyl alcohol	7,838	[88]	1,000	999	79, 107, 122
	Benzyl alcohol	1,563	[86]	1,000	1,035	51, 79, 108
10	Ethyl n-octanoate	2,124	[88]	2,000	1,723	101, 127
	1-Hexanol	2,200	[88]	2,000	1,604	56, 84
	Ethyl Hexanoate	2,356	[88]	2,000	1,723	70, 99
11	Ethyl hexadecanoate[3]	600 – 2,410	[91]	115[2]	1901	55, 83, 97
12	Isoamyl alcohol[3]	80,000 – 300,000	[92]	1,500[2]	23,085	42,55, 70
	Ethyl lactate[3]	56,800 – 80,900	[93]	1,500[2]	30,372	29, 45, 75
13	Isobutanol[3]	25,000 - 86,000	[92]	1,500[2]	22,800	33, 43

[1]Compound was not detected in actual wine treatments for the present study.
[2]Due to high concentration in wines stock solution process was modified to a single solution of 50 μL added to 250 mL of 95% Ethanol solution.
[3]Compound was analyzed using solid-phase microextraction.
